# Editorial: Organelles Relationships and Interactions: A Cancer Perspective

**DOI:** 10.3389/fcell.2021.678307

**Published:** 2021-04-08

**Authors:** Simone Patergnani, Saverio Marchi, Benjamin Delprat, Mariusz R. Wieckowski

**Affiliations:** ^1^Section of Experimental Medicine, Laboratory for Technologies of Advanced Therapies (LTTA), Department of Medical Sciences, University of Ferrara, Ferrara, Italy; ^2^Department of Clinical and Molecular Sciences, Marche Polytechnic University, Ancona, Italy; ^3^MMDN, Univ Montpellier, EPHE, INSERM, Montpellier, France; ^4^Laboratory of Mitochondrial Biology and Metabolism, Nencki Institute of Experimental Biology of the Polish Academy of Sciences, Warsaw, Poland

**Keywords:** cancer, organelles, endoplasmic reticulum, mitochondria, lysosomes, therapy

## Introduction

For several decades, cellular organelles have been considered as membrane-limited independent structures or static units, with defined composition and specialized functions. Nowadays, it is widely accepted that intracellular organelles are dynamic entities that interact and communicate with each other through several types of membrane contacts. The first evidence was obtained in 1959, when by using electron microscopy a close proximity between the endoplasmic reticulum (ER) and mitochondria has been observed (Copeland and Dalton, 1959). Now, organelle communication represents a common phenomenon, involving mitochondria, ER, plasma membrane (PM), lysosomes, nucleus, Golgi apparatus, and peroxisomes (Bravo-Sagua et al., 2014).

Moreover, thanks to the significant advances in the research field, it has been demonstrated that organelles not only communicate *via* contact sites but also create specialized sub-compartments, such as the mitochondria-associated membranes (MAMs), which are the result of the close interaction mostly between mitochondria and ER (Giacomello and Pellegrini, 2016; Annunziata et al., 2018).

Intracellular organelle crosstalk is fundamental for all biological functions, including metabolism, organelle integrity, immune response, regulation of cell death, and response to external environmental signals (Gottschling and Nystrom, 2017). Any change in this extensive communication provokes alterations of the cellular microenvironment and activates relevant signaling cascades that affect proper cellular functions and influence cellular survival. It is not surprising that emerging evidence has highlighted the failure of organelle dynamics and communication in various human diseases, especially in cancer (Giorgi et al., 2018; Patergnani et al., 2020).

Consistently, in this pathological context emerging studies have associated changes in inter-organelle communication with the most salient characteristics of cancer: cell survival, proliferation, and adaption to stressed conditions, such as reduced oxygen and nutrients availability and exposure to therapeutics.

This Research Topic entitled “***Organelles Relationships and Interactions: a Cancer*** ***Perspective***” aims to represent an updated showcase of the current knowledge about the intracellular relationship among several organelles, depict their relevance for cell death and survival, and analyze their contribution in cancer. The published original research and review articles are briefly described below:

- **Diaz et al**., focused on MAMs and on the proteins that reside at the ER-mitochondria interphase, describing their role in cancer disease progression. Interestingly, they also analyzed the contribution of ER-mitochondria interactions with other organelles, such as lysosomes and peroxisomes.
- **Madreiter-Sokolowski et al**., demonstrated that cancer cells use a signaling axis composed by UCP2 and Rab32 to control the ER-mitochondrial tethering, regulate the mitochondrial metabolism and down-regulate the Calcium (Ca^2+^)-mediated cell death. Consistently, they found important correlations between proteins stabilizing mitochondrial-ER linkage and UCP2 in human cancer tissues.
- **Gil-Hernandez et al**., reviewed the state-of-the-art of the importance of the membrane contact sites (MCS) to regulate the Ca^2+^ and lipid signaling in cancer cells and in tumor progression. Specifically, they described the impact of specific proteins associated to MCS and reported the interactions between ER–PM, ER–mitochondria, and ER–Golgi.
- **Halcrow et al**., described the importance of acid-base microenvironment and balance on cancer cell survival. They also discussed potential mechanisms underlying acid/base-dependent anti-cancer effects of agents that regulate the acid-base balance of microenvironment. Particularly, they focused their attention on compounds that de-acidify lysosomes to inhibit autophagosome lysosome fusion or drugs de-acidifying Golgi apparatus and secretory vesicles to affect secretion.
- **Ahumada-Castro et al**., emphasized the role of inositol 1,4,5-trisphosphate (IP3) receptors (IP3Rs), in the inter-organelle communications, with particular attention to the relationship between mitochondria ER and lysosomes, and how crosstalk of these organelle impact the intracellular signaling pathway associated with cancer growth. In particular, they focused on the critical role of the IP3Rs-mediated Ca^2+^ release and its possible implications in cancer.
- **Yu et al**., explored the current knowledge about the composition and functions of the MAMs and how MAMs-resident proteins contribute to carcinogenesis by regulating the cell death event. In particular, the authors discussed the emerging evidence showing the critical roles of MAMs in breast cancer, one of the most common cancers in the world.
- **Amodio et al**., reviewed the important interconnections that exist between different components of the unfolded protein response and ER-mitochondria contact sites in regulating cell survival and death and how they impact on tumor development. Authors focused their attention to PERK, IRE1alpha, and diverse ER chaperones and ER oxidoreductases, which are notably involved in regulation of the ER-mitochondria contact sites, as well as in tumor growth.
- **Machado et al**., reported the latest advances in the knowledge of the role of the lysosomal compartment during cancer progression. Consistently, authors described how changes in lysosomal biogenesis and functioning are critical from a perspective of acidic tumor microenvironment as well as their capacity to communicate with the other intracellular organelle.

## Perspectives

Emerging insights not only continuously demonstrate that intracellular organelles communicate one with the other. It is clear that these communications also affect several aspects of cancer features, thus playing a critical role in tumor development, growth, and metastasis. However, despite these significant improvements, we can only affirm to have unmasked a little fragment concerning the impact of inter-organelle crosstalk in tumorigenesis. To date, it remains unclear whether the alterations observed in the organelle communication are simple a cause or consequence of cancer. Further investigations are required to extend our knowledge in this field and to conceive a therapeutic approach against cancers built on the modulation of organelle interactions.

## Author Contributions

SP drafted the manuscript that was reviewed and edited by SM, BD, and MRW. All authors co-edited the Research Topic.

## Conflict of Interest

The authors declare that the research was conducted in the absence of any commercial or financial relationships that could be construed as a potential conflict of interest.
